# Impact of stenosis resistance and coronary flow capacity on fractional flow reserve and instantaneous wave-free ratio discordance: a combined analysis of DEFINE-FLOW and IDEAL

**DOI:** 10.1007/s12471-023-01796-x

**Published:** 2023-08-18

**Authors:** Valérie Stegehuis, Coen Boerhout, Yuetsu Kikuta, Maribel Cambero-Madera, Niels van Royen, Hitoshi Matsuo, Masafumi Nakayama, Guus de Waard, Paul Knaapen, Sukhjinder Nijjer, Ricardo Petraco, Maria Siebes, Justin Davies, Javier Escaned, Tim van de Hoef, Jan Piek

**Affiliations:** 1 Heart Centre, Amsterdam Cardiovascular Sciences, Amsterdam UMC—location AMC, Department of Cardiology, University of Amsterdam, Amsterdam, The Netherlands; 2 Imperial College London, London, UK; 3 Tergooi Hospital, Blaricum, The Netherlands; 4 Department of Cardiology, Radboud University Nijmegen, Nijmegen, The Netherlands; 5 Gifu Heart Center, Gifu, Japan; 6 Toda Chuo General Hospital, Toda, Japan; 7 Amsterdam UMC—location VUMC, Department of Cardiology, Heart Centre, Amsterdam Cardiovascular Sciences, VU University, Amsterdam, The Netherlands; 8 Department of Biomedical Engineering and Physics, Amsterdam UMC—location AMC, Amsterdam, The Netherlands; 9 Hospital Clinico San Carlos IDISSC, Complutense University, Madrid, Spain

**Keywords:** Instantaneous wave-free ratio, Fractional flow reserve, Coronary flow reserve, Coronary flow capacity

## Abstract

**Background:**

The pressure-derived parameters fractional flow reserve (FFR) and the emerging instantaneous wave-free ratio (iFR) are the most widely applied invasive coronary physiology indices to guide revascularisation. However, approximately 15–20% of intermediate stenoses show discordant FFR and iFR, and therapeutical consensus is lacking.

**Aims:**

We sought to associate hyperaemic stenosis resistance index, coronary flow reserve (CFR) and coronary flow capacity (CFC) to FFR/iFR discordance.

**Methods:**

We assessed pressure and flow measurements of 647 intermediate lesions (593 patients) of two multi-centre international studies.

**Results:**

FFR and iFR were discordant in 15% of all lesions (97 out of 647). FFR+/iFR− lesions had similar hyperaemic average peak velocity (hAPV), CFR and CFC as FFR−/iFR− lesions, whereas FFR−/iFR+ lesions had similar hAPV, CFR and CFC as FFR+/iFR+ lesions (*p* > 0.05 for all). FFR+/iFR− lesions were associated with lower baseline stenosis resistance, but not hyperaemic stenosis resistance, compared with FFR−/iFR+ lesions (*p* < 0.001).

**Conclusions:**

Discordance with FFR+/iFR− is characterised by maximal flow values, CFR, and CFC patterns similar to FFR−/iFR− concordance that justifies conservative therapy. Discordance with FFR−/iFR+ on the other hand, is characterised by low flow values, CFR, and CFC patterns similar to iFR+/FFR+ concordance that may benefit from percutaneous coronary intervention.

**Supplementary Information:**

The online version of this article (10.1007/s12471-023-01796-x) contains supplementary material, which is available to authorized users.

## What’s new?

- Discordance with abnormal FFR and normal iFR is characterised by maximal flow values, similar to concordant normal FFR and iFR lesions.
- In patients with abnormal FFR and normal iFR, conservative therapy may be justified.
- Discordance with normal FFR and abnormal iFR is characterised by low flow values, similar to concordant abnormal FFR and iFR values.
- Patients with normal FFR and abnormal iFR may benefit from intervention.

## Introduction

The accuracy of coronary angiography (CAG) in assessing functional stenosis significance is poor [1, 2], and additional coronary physiology assessment improves the identification of haemodynamically relevant stenosis [3, 4]. Among these are the coronary pressure-derived fractional flow reserve (FFR) and instantaneous wave-free ratio (iFR). Both indices are translesional pressure ratios, but FFR is calculated from averaged whole cycle hyperaemic pressure measurements, while iFR is calculated from selective diastolic non-hyperaemic pressure measurements. Disagreement occurs in up to 20% of vessels [5–7], but comparisons with independent reference standards have documented equivalent diagnostic efficiency between the two techniques [8]. In addition, two large randomised clinical trials reported non-inferiority of iFR-guided intervention to FFR-guided intervention with respect to adverse cardiovascular events [9, 10]. Interestingly, revascularisation rates were lower in the iFR-guided strategy arm, but no difference in 1‑year major cardiac event (MACE) rates was apparent between the two strategies [11]. With FFR and iFR now used interchangeably in clinical practice, or even together in the same patient, the frequent discordance and intriguing findings on revascularisation rates warrant detailed insights into disagreement between the techniques. Previous studies have suggested that measurements of coronary flow reserve (CFR) can provide valuable insight into the origin of iFR/FFR discordance [12–14], but comprehensive physiological assessment using basal and hyperaemic stenosis resistance index, stenosis-specific markers of physiological severity, and coronary flow capacity (CFC), a comprehensive myocardial perfusion marker, may allow more detailed insight into the origin and consequences of iFR/FFR discordance. Therefore, we sought to describe the origin of iFR/FFR disagreement with respect to physiological stenosis severity and myocardial perfusion impairment to guide the combined use of iFR and/or FFR in clinical practice.

## Methods

### Patient population

This analysis included patients from two international multi-centre studies on comprehensive invasive physiological stenosis assessment: the IDEAL registry, and the DEFINE-FLOW study (NCT02328820). Rationale and design of DEFINE-FLOW [15] and the results of IDEAL [16] have been published elsewhere. From DEFINE-FLOW, solely measurements approved by the core laboratory were used in this subanalysis, since the required physiological data for hyperaemic stenosis resistance and CFC calculations were only available in cases in which core lab data were reported.

### Cardiac catheterisation and physiological assessment

Intracoronary nitroglycerin (100–300 µg) was administered at the beginning of the procedure, and repeated every 30 min if necessary. After diagnostic coronary angiography, a 0.014′′ dual pressure and Doppler flow velocity sensor tipped guidewire (ComboWire XT; Philips Volcano, San Diego, CA) was zeroed to atmospheric pressure, and subsequently calibrated to aortic pressure at the ostium of the guiding catheter. Afterwards, the guidewire was positioned at least five vessel diameters distal to the lesion. After obtaining a stable flow signal, wire position was recorded fluoroscopically and adenosine was administered by an intracoronary bolus injection of 60–150 µg, or intravenous infusion of adenosine at a dose of 140 μg/kg/min to induce hyperaemia [17, 18]. After obtaining the measurements, the guidewire was pulled back to the guiding catheter to assess pressure drift.

FFR was calculated as the mean distal to aortic pressure at peak hyperaemia. CFR was calculated as the ratio of hyperaemic (hAPV) to baseline (bAPV) average peak flow velocity. iFR was calculated by dividing distal resting pressure by aortic pressure in the diastolic wave-free period. Baseline stenosis resistance was calculated as the ratio of mean trans-stenotic pressure gradient to bAPV with a cut-off value of 0.66 [19], whereas hyperaemic stenosis resistance was calculated as the ratio of mean trans-stenotic pressure gradient to hAPV, with a cut-off value of 0.80 [20]. Binary iFR ≤ 0.89, FFR ≤ 0.8, and hyperaemic stenosis resistance ≥ 0.8 mm Hg/cm/s were considered abnormal. Normal CFC was defined as a CFR ≥ 2.8 and an hAPV of ≥ 49.0 cm/s. Mildly reduced CFC was defined as a CFR < 2.8 but > 2.1 and an hAPV of < 49.0 but > 33.0 cm/s. Moderately reduced CFC was defined as a CFR ≤ 2.1 and > 1.7, and an hAPV of ≤ 33.0 and > 26.0 cm/s. Finally, severely reduced CFC was defined as a CFR ≤ 1.7, and an hAPV of ≤ 26.0 cm/s [21]. Abnormal CFC was defined as a moderately to severely reduced CFC.

### Statistical analysis

All analyses were performed at the lesion level, except for baseline patient characteristics. Continuous data were presented as mean ± standard deviation or median (first, third quartile [Q1, Q3]), and were compared by using the paired Kruskal-Wallis test. Analyses across iFR/FFR concordance and discordance groups were compared with 1‑way ANOVA, Kruskal–Wallis, χ^2^ or Fisher’s exact test. Receiver operating characteristic (ROC) curves were computed to compare the diagnostic efficiency of each invasive physiological index against severely or moderately reduced CFC by the area under the ROC curve (ROC_AUC_). ROC_AUC_ was calculated using DeLong’s method. Using the clinically established FFR and iFR cut-off values (≤ 0.80 for FFR and ≤ 0.89 for iFR), diagnostic agreement, sensitivity, specificity, positive predictive value, and negative predictive value were evaluated between FFR and iFR. Applicable tests were 2‑tailed and *p* < 0.05 was considered statistically significant. For all statistical analyses, the STATA version 15.1 (StataCorp, College Station, Texas) software package was used.

## Results

A total of 593 patients with 647 lesions were analysed; 281 patients with 281 measurements from IDEAL, and 312 patients with 366 measurements from DEFINE FLOW. Mean age was 65 ± 10 years, and 77% of patients were male (Tab. 1). Physiologic and angiographic characteristics are summarised in Tab. 2.

**Table 1:** Baseline characteristics

*Demographics*		
Age (years)	65 ± 10	
Male	447	77%
*Risk factors and medical history*		
Hypertension	357	61%
Dyslipidaemia	449	76%
Family history	228	40%
Smoking (current)	215	37%
Diabetes	156	26%
Renal dysfunction	31	6%
Prior MI	126	21%
Prior PCI	131	20%
Peripheral vascular disease	18	3%
Cerebrovascular disease	29	4%
*Medications*		
Aspirin	421	72%
Other anti-platelet	209	68%
Anti-coagulant	32	10%
Beta-blocker	316	54%
Calcium antagonist	162	28%
Nitrates	145	47%
Statin	381	65%
Other lipid drugs	16	5%
RAAS antagonist	204	35%
Diuretic	90	17%
Anti-diabetics (all)	49	16%
Insulin	11	4%

Values are reported as *n* (%) or mean ± SD

*MI* myocardial infarction, *PCI* percutaneous coronary intervention, *RAAS* renin-angiotensin-aldosterone system, *SD* standard deviation

**Table 2:** Physiologic and angiographic characteristics

LV ejection fraction (%)	60 [58, 65]	
Visual diameter stenosis (%)	60 [50, 70]	
Prior MI	54	8%
Prior PCI	51	14%
In-stent lesion	13	4%
Coronary distribution		
– LAD	393	61%
– LCX	131	20%
– RCA	123	19%
Adenosine route		
– Intracoronary	488	75%
– Intravenous	159	25%
FFR	0.83 [0.74, 0.89]	
CFR	2.1 [1.7, 2.7]	
iFR	0.92 [0.85, 0.96]	

Values are reported as *n* or median [Q1, Q3]

Reported percentages are excluding missing values

*LV* left ventricular, *MI* myocardial infarction, *PCI* percutaneous coronary intervention, *LAD* left anterior descending artery, *LCX* left circumflex artery, *RCA* right coronary artery, *FFR* fractional flow reserve, *CFR* coronary flow reserve, *iFR* instantaneous wave-free ratio

### Agreement between FFR and iFR

**Fig. 1:**
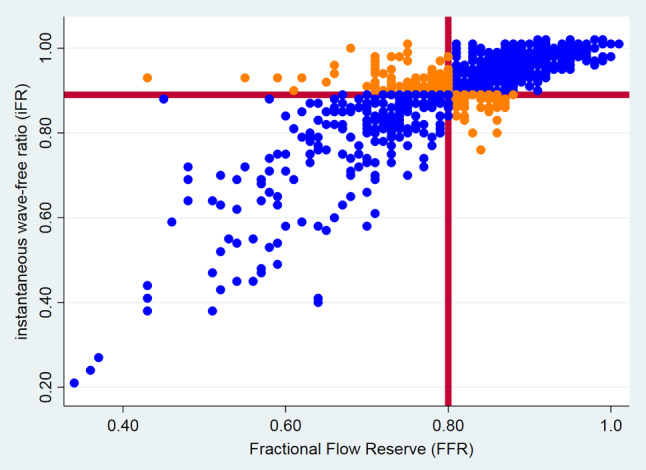
iFR-FFR discordance. The *highlighted red lines* represent the cut-off values for FFR (≤ 0.80) and iFR (≤ 0.89). Concordant cases are *coloured blue*, discordant cases are *coloured red* (*FFR* fractional flow reserve, *iFR* instantaneous wave-free ratio)

Patient characteristics of FFR/iFR discordant groups are shown in Tab. 3. FFR and iFR measurements were discordant in 15% (*n* = 97) cases, comprising of 57% (*n* = 55) lesions with FFR+/iFR− and 43% (*n* = 42) lesions with FFR−/iFR+ (Fig. 1). Patients in the FFR+/iFR− group were younger (*p* = 0.02) and were more frequently active smokers (*p* = 0.04), whereas patients with FFR−/iFR+ were more frequently diabetic (*p* = 0.08). For the left anterior descending artery (LAD), a total of *n* = 60 (15%) lesions (*n* = 30 [FFR+/iFR−] and *n* = 30 [FFR−/iFR+]) were discordant. For the right circumflex artery (RCX), a total of *n* = 26 (20%) lesions (*n* = 19 [FFR+/iFR−] and *n* = 7 [FFR−/iFR+]) were discordant. For the right coronary artery (RCA), a total of *n* = 11 (9%) lesions (*n* = 6 [FFR+/iFR−] and *n* = 5 [FFR−/iFR+]) were discordant. Thus, discordance occurred most frequently in the RCX.

**Table 3:** Study population characteristics of the FFR/iFR discordant lesions groups

	FFR+/iFR− lesion group	FFR−/iFR+ lesion group	*p*-value
Patients	47 (8%)	40 (7%)	
Lesions	55 (9%)	42 (6%)	
Age (years)	62 ± 10	66 ± 10	0.02*
Male	38 (84)	27 (68)	0.321
Hypertension	28 (60)	22 (55)	0.105
Dyslipidaemia	40 (85)	30 (75)	0.701
Smoking (current)	17 (36)	9 (23)	0.04*
Diabetes	9 (19)	16 (40)	0.08
Renal dysfunction	2 (6)	2 (14)	0.631
Prior MI	11 (23)	8 (20)	0.813
Prior PCI	16 (47)	7 (50)	0.979
Peripheral vascular disease	2 (6)	0 (0)	0.429
Cerebrovascular disease	1 (3)	2 (14)	0.221
Coronary artery			
– LAD (*n* = 393)	30	30	
– RCX (*n* = 131)	19	7	
– RCA (*n* = 123)	6	5	

Values are *n* (%) or mean ± SD

*MI* myocardial infarction, *PCI* percutaneous coronary intervention, *LAD* left anterior descending artery, *RCX* right circumflex artery, *RCA* right coronary artery, *FFR* fractional flow reserve, *iFR* instantaneous wave-free ratio, *SD* standard deviation

*p-value < 0.05

### Association between iFR, FFR and baseline and hyperaemic flow

**Fig. 2:**
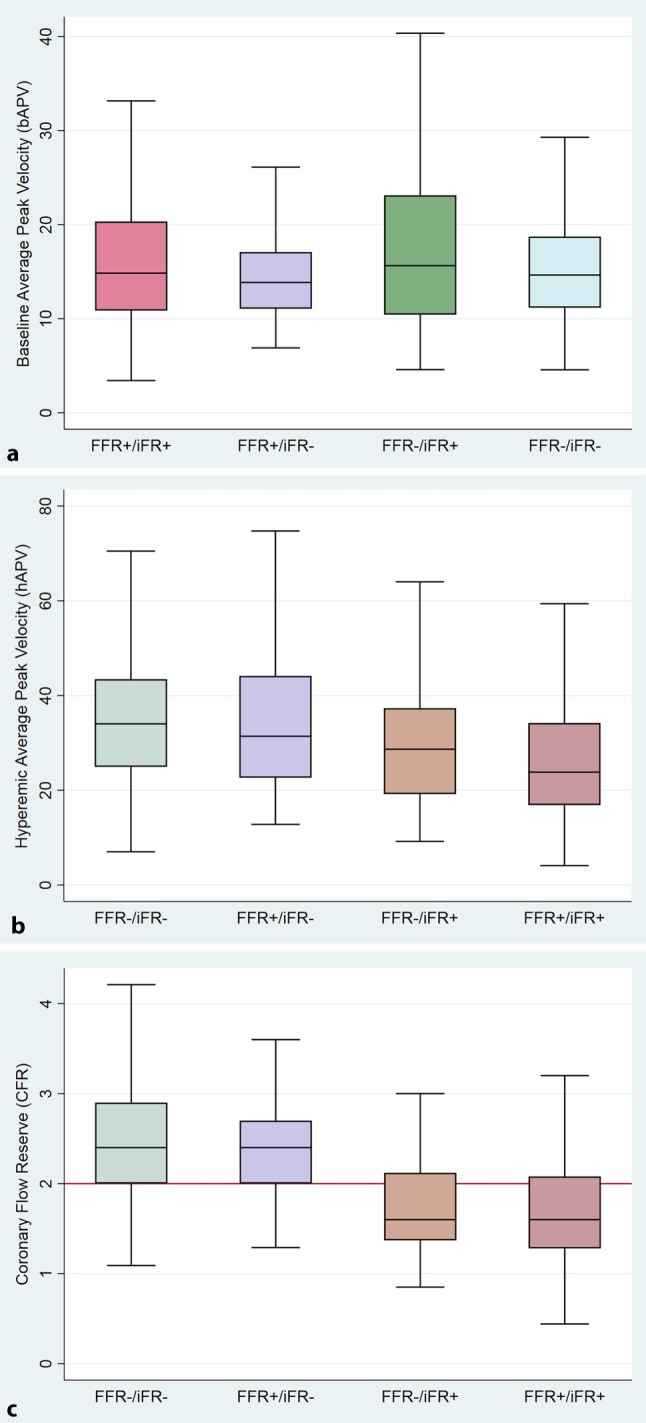
Boxplots of bAPV (**a**), hAPV (**b**) and CFR (**c**) for each FFR/iFR group (*FFR/iFR* fractional flow reserve/instantaneous wave-free ratio)

Lesion and physiology characteristics across FFR/iFR groups are summarised in Tab. S1 in ESM. bAPV and hAPV were not significantly different between the discordant groups (*p* = 0.202 and *p* = 0.09 respectively) (Fig. 2a). CFR was significantly different across groups (*p* = 0.0001). Lesions with FFR+/iFR− discordance had similar hAPV and CFR compared with lesions with FFR−/iFR− concordance (hAPV 31 cm/s [Q1, Q3: 23, 44] and CFR 2.4 [Q1, Q3: 2.0, 2.7] versus hAPV 34 cm/s [Q1, Q3: 25, 44] and CFR 2.4 [Q1, Q3: 2.0, 2.9] respectively [*p* > 0.05 for all]). In contrast, lesions with FFR−/iFR+ discordance had similar hAPV and CFR compared with lesions with FFR+/iFR+ concordance (hAPV 29 cm/s [Q1, Q3: 19, 37] and CFR 1.6 [Q1, Q3: 1.4, 2.1] versus hAPV 24 cm/s [Q1, Q3: 17, 34] and CFR 1.6 [Q1, Q3: 1.3, 2.1] [*p* < 0.001 for all]) (Fig. 2b,c).

### iFR, FFR, CFC and stenosis resistance across FFR/iFR groups

**Fig. 3:**
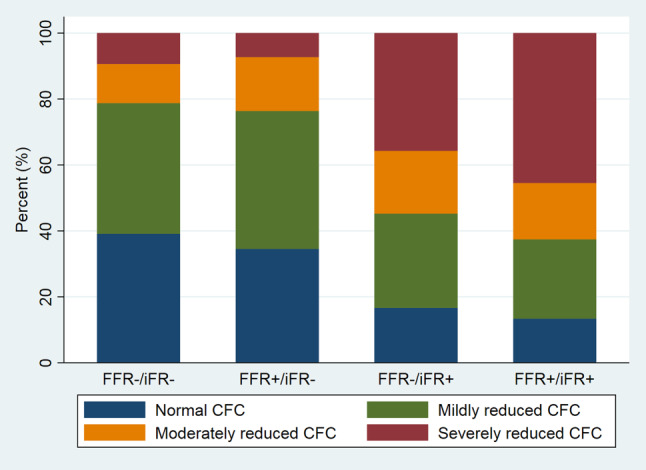
Prevalence of CFC categories for each FFR/iFR group (*FFR* fractional flow reserve, *iFR* instantaneous wave-free ratio, *CFC* coronary flow capacity)

Figure 3 shows the distribution of lesions across the CFC categories within each of the FFR/iFR groups. Lesions with FFR+/iFR− discordance had abnormal CFC in 22% of cases, similar to lesions with FFR−/iFR− concordance where 21% of cases had abnormal CFC (*p* = 0.64). Lesions with FFR−/iFR+ discordance had abnormal CFC in 55% of cases, similar to lesions with FFR+/iFR+ concordance where 63% of lesions had abnormal CFC (*p* = 0.28).

In ROC analysis, iFR showed better diagnostic efficiency than FFR for the identification of abnormal CFC (ROC_AUC_: 0.74 versus 0.68 respectively: *p* < 0.001) (see Fig. S1 in ESM).

Baseline stenosis resistance was not significantly different between the discordant groups (*p* = 0.093), but hyperaemic stenosis resistance was 0.70 (0.50, 0.96) for FFR+/iFR− lesions versus 0.42 (0.36, 0.63) for FFR−/iFR+ lesions (*p* < 0.001). Baseline stenosis resistance was highest for vessels with FFR−/iFR+ discordance and hyperaemic stenosis resistance was highest for vessels with FFR+/iFR− discordance. Moreover, there was a significant difference in CFR and delta APV across the FFR/iFR groups and across the discordance groups specifically: delta APV 18 (13, 24) and CFR 2.4 (2.0, 2.7) for the FFR+/iFR− group versus delta APV 11 (6, 17) and CFR 1.6 (1.4, 2.1) for the FFR−/iFR+ group (*p* < 0.001 for delta APV and *p* < 0.001 for CFR) (see Tab. S1 and Fig. S2 in ESM).

In vessels with FFR+/iFR− discordance, iFR values were close to the cut-off value (iFR 0.91 [Q1, Q3: 0.90, 0.93] versus FFR 0.75 [Q1, Q3: 0.73, 0.77]). Similarly, in vessels with FFR−/iFR+ discordance, iFR values were close to the cut-off value (iFR 0.87 [Q1, Q3: 0.84, 0.88] versus FFR 0.84 [Q1, Q3: 0.82, 0.86]) (Tab. S1 in ESM).

Vessels with FFR+/iFR− discordance had similar baseline stenosis resistance compared with vessels with FFR−/iFR+ discordance (0.45 mm Hg/cm/s [Q1, Q3: 0.31, 0.63] versus 0.59 mm Hg/cm/s [Q1, Q3: 0.35, 0.76] respectively; *p* = 0.093). In contrast, vessels with FFR+/iFR− discordance had higher hyperaemic stenosis resistance compared with vessels with FFR−/iFR+ discordance (hyperaemic stenosis resistance 0.70 mm Hg/cm/s [Q1, Q3: 0.50, 0.96] versus 0.42 mm Hg/cm/s [Q1, Q3: 0.36, 0.63] respectively; *p* < 0.001) (see Fig. S2 in ESM).

## Discussion

The main finding of this study is that discordance between FFR and iFR, in terms of stenosis severity classification, is explained by the magnitude in maximal flow in the evaluated coronary artery. Discordance due to abnormal FFR is characterised by maximal flow values, CFR, and CFC patterns similar to those found in lesions with concordantly normal measurements. In contrast, discordance due to normal FFR is characterised by low flow values, CFR, and CFC patterns similar to those found in lesions with concordantly abnormal measurements.

### Impact of coronary flow and stenosis resistance on FFR/iFR discordance

Similar to previous reports, discordance between FFR and iFR occurred in 15% of cases in the current analysis [6]. As depicted in Tab. S1 in ESM, the main difference between the discordance groups is reflected by the delta APV, CFR and hyperaemic stenosis resistance. This indicates the direct relationship of the discordance between FFR and iFR to the delta APV per interrogated vessel and the proportionally linear relationship between pressure and flow across a stenosis [22]. This relationship can be described by the quadratic equation of the form $\Updelta \mathrm{P}=\mathrm{Av}+\mathrm{B}v^{2}$; where $\Updelta \mathrm{P}$ is the pressure drop across the stenosis, v is the flow velocity and A and B are stenosis-specific coefficients. As such, the pressure drop is quadratically dependent of the flow through it and this could explain the differences between the discordance groups. In the FFR+/iFR− group, the intermediate lesions are characterised by a higher epicardial resistance (hyperaemic stenosis resistance) and increased CFR as a larger delta APV results in a larger pressure drop across that lesion during hyperaemia compared to the resting conditions, and thus, an abnormal FFR in the presence of a normal iFR. In comparison, within the FFR−/iFR+ group, the delta APV is limited and together with a lower hyperaemic stenosis resistance results in a limited pressure drop during hyperaemia compared with that at rest, resulting in a normal FFR in the presence of an abnormal iFR (Tab. S1 in ESM).

### Comparison with previous studies

The present study confirms, and expands on, previous studies on flow characteristics in FFR/iFR discordance. Petraco et al. [23] and Cook et al. [5] were the first to provide insights in the origin of FFR/iFR discordance. The shared finding of these studies was that vessels with FFR+/iFR− have higher hyperaemic flow and CFR compared with FFR−/iFR+ vessels. Our findings provide further evidence for the role of maximal flow values in FFR/iFR discordance (Tab. S1 in ESM). Patients in the present study with FFR−/iFR+ discordance were significantly older than patients with FFR+/iFR− discordance (mean age 66 ± 10 versus 62 ± 10; *p* = 0.02) and tended to have a higher prevalence of diabetes (*p* = 0.08). Increasing age and diabetes are associated with a diminished response to a potent vasodilator, consequently leading to lower maximal flow values, impacting FFR/iFR discordance as described above [1, 24, 25]. Since non-hyperaemic stenosis physiology, assessed by baseline stenosis resistance, was similar across the two discordance patterns, the physiological origin of FFR/iFR discordance lies in hyperaemic vessel flow characteristics that are not related to stenosis severity. This is further supported by the distribution of CFC across the FFR/iFR groups (Fig. 3).

High hyperaemic flow and CFR are predictors of benign long-term clinical outcomes, even when FFR ≤ 0.80 [14]. The previously reported reports from the DEFINE-FLOW study and the ILIAS-registry indicated that patients with vessels with normal CFR and abnormal FFR in whom revascularisation was deferred, have outcomes similar to those patients who were treated with revascularisation [26]. Moreover, low CFR is independently associated with poor long-term clinical outcome [12, 27–29]. Hence, iFR seems efficient in identifying those stenoses that are associated with impaired flow characteristics since the current data confirm in detail that FFR/iFR discordance occurs on the basis of variable maximal flow values that are generally more benign in iFR− vessels without differences in stenosis severity. In this population, this efficiency was not increased by additional measurement of FFR. These findings should trigger further evaluation of the prognostic impact of FFR/iFR discordance, on which evidence remains scarce [30]. From the comprehensive perspective of coronary haemodynamics provided by CFC, FFR/iFR discordance due to abnormal FFR is associated with normal or mildly reduced CFC in nearly 80% of cases. This may explain the lower revascularisation rates noted in the iFR arms of the DEFINE-FLAIR and iFR SWEDEHEART which, as noted above, were not associated with worse outcomes than the FFR-based strategy. Alternatively, FFR/iFR discordance due to normal FFR might look worrisome: around 50% of these cases have moderately or severely reduced CFC. The prognostic implications of this finding have been highlighted in previous works [21]. It remains unclear if revascularisation of stenosis included in this category would be associated with improved patient outcomes. Moreover, other causes of iFR/FFR discordance have been suggested: lesion location and severity, heart rate, age and beta blocker use affect mainly FFR and should be taken into account [31]. It was suggested that non-hyperaemic indices may be less reliable in proximal LAD lesions, since these supply a large amount of subtended myocardial mass [32]. However, this physiological consideration is based on the relationship between larger subtended myocardial mass and higher maximal hyperaemic flow across the stenosis, which leads to higher pressure gradients across a given stenosis. As discussed above, this phenomenon is indeed associated with discordance between iFR and FFR, but also with benign coronary flow characteristics. This is supported by clinical outcome data from a combined analysis of DEFINE-FLAIR and iFR-SWEDEHEART as well, documenting a lower incidence of adverse events for LAD lesions deferred based on iFR measurements compared with LAD lesions deferred based on FFR measurements.

### Clinical implications

In clinical practice, borderline iFR values will be followed by FFR measurements for clinical decision-making. In general, a borderline iFR value with an abnormal FFR value will be interpreted as an indication for percutaneous coronary intervention, while percutaneous coronary intervention will be postponed in case of a normal FFR. The present study, including the ROC analysis comparing iFR and FFR, indicates an opposite interpretation: a patient may benefit from an intervention in case of iFR+/FFR−, while conservative therapy may be justified in case of iFR−/FFR+. In case of doubt, performing CFR measurements may provide a more robust answer whether it is safe to defer a certain lesion of intermediate severity, since hyperaemic flow is significantly different between FFR+/iFR− and FFR−/iFR+ lesions, where FFR+/iFR− lesions are associated with higher CFR and thus benign long-term clinical outcomes.

### Limitations

The total number of discordant iFR/FFR lesions in the DEFINE-FLOW and IDEAL is small, but these two studies combined provide the largest multi-centre analysis of patients with intermediate coronary lesions undergoing invasive physiological interrogation by combined pressure and flow velocity measurements. Second, clinical follow-up after coronary physiological assessment was not routinely performed in IDEAL, prohibiting the evaluation of clinical outcomes.

## Conclusion

Discordance between iFR and FFR is an inevitable phenomenon, occurring in 15% of cases in this analysis, and is explained by the magnitude in maximal flow in the evaluated coronary artery unrelated to stenosis severity.

### Supplementary Information

**Fig. S1** Area under the receiver operating characteristic curve (ROC_auc_) for FFR and iFR against abnormal CFC (*FFR* fractional flow reserve, *iFR* instantaneous wave-free ratio, *CFC* coronary flow capacity)

**Fig. S2** BSR and HSR over FFR/iFR groups (*FFR/iFR* fractional flow reserve/instantaneous wave-free ratio, *BSR* basal stenosis resistance, *HSR* hyperaemic stenosis resistance)

**Tab. S1** Lesion and physiology characteristics per group
