# Supplementary figures and images for: Impact of stenosis resistance and coronary flow capacity on fractional flow reserve and instantaneous wave-free ratio discordance: a combined analysis of DEFINE-FLOW and IDEAL
Source: Neth Heart J. 2023 Aug 18;31(11):434–43. doi: 10.1007/s12471-023-01796-x (PMC10602988; doi:10.1007/s12471-023-01796-x)

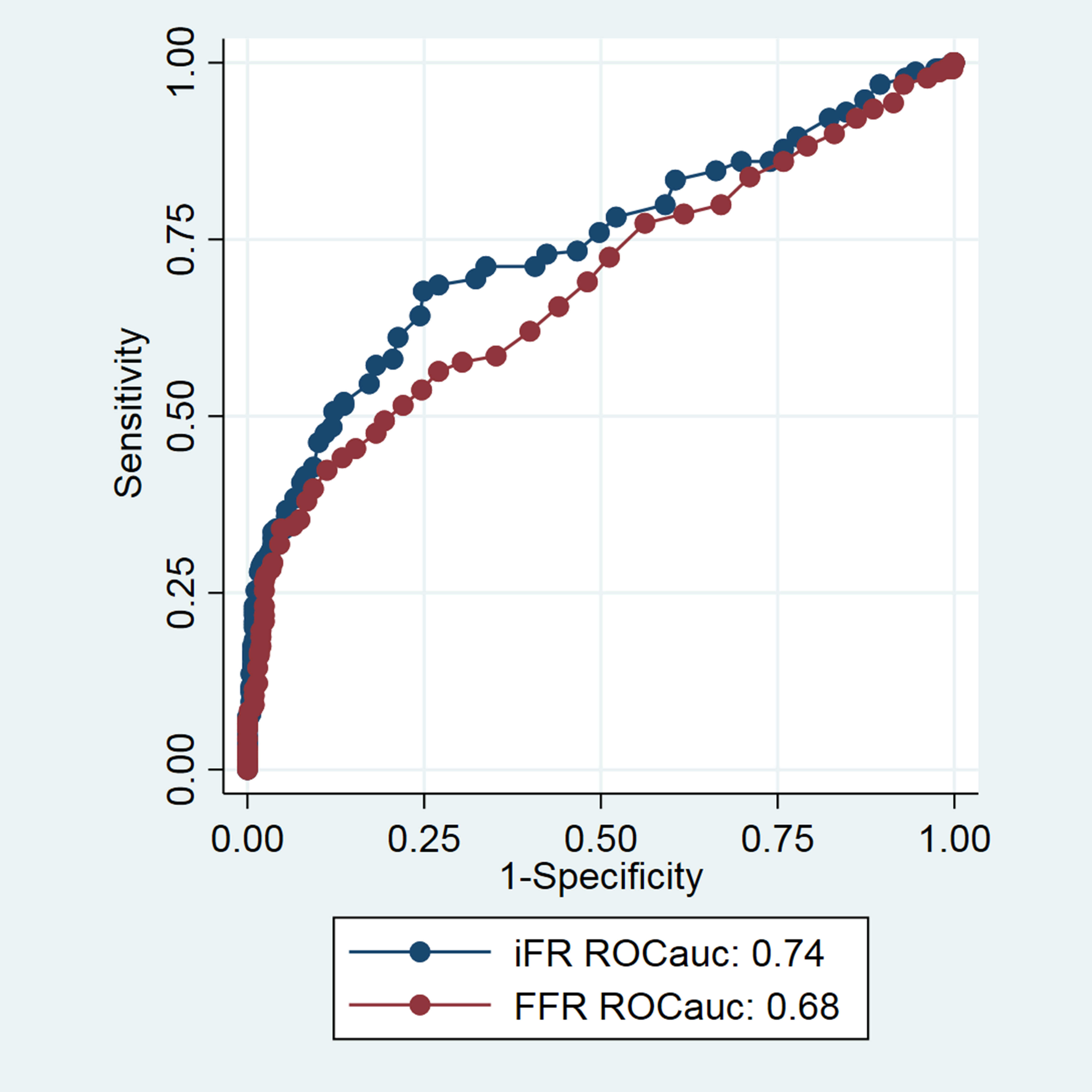

Supplement: Supplementary file 1 — Fig. S1 Area under the receiver operating characteristic curve (ROCauc) for FFR and iFR against abnormal CFC (FFR fractional flow reserve, iFR instantaneous wave-free ratio, CFC coronary flow capacity) [file 12471_2023_1796_MOESM1_ESM.tif]

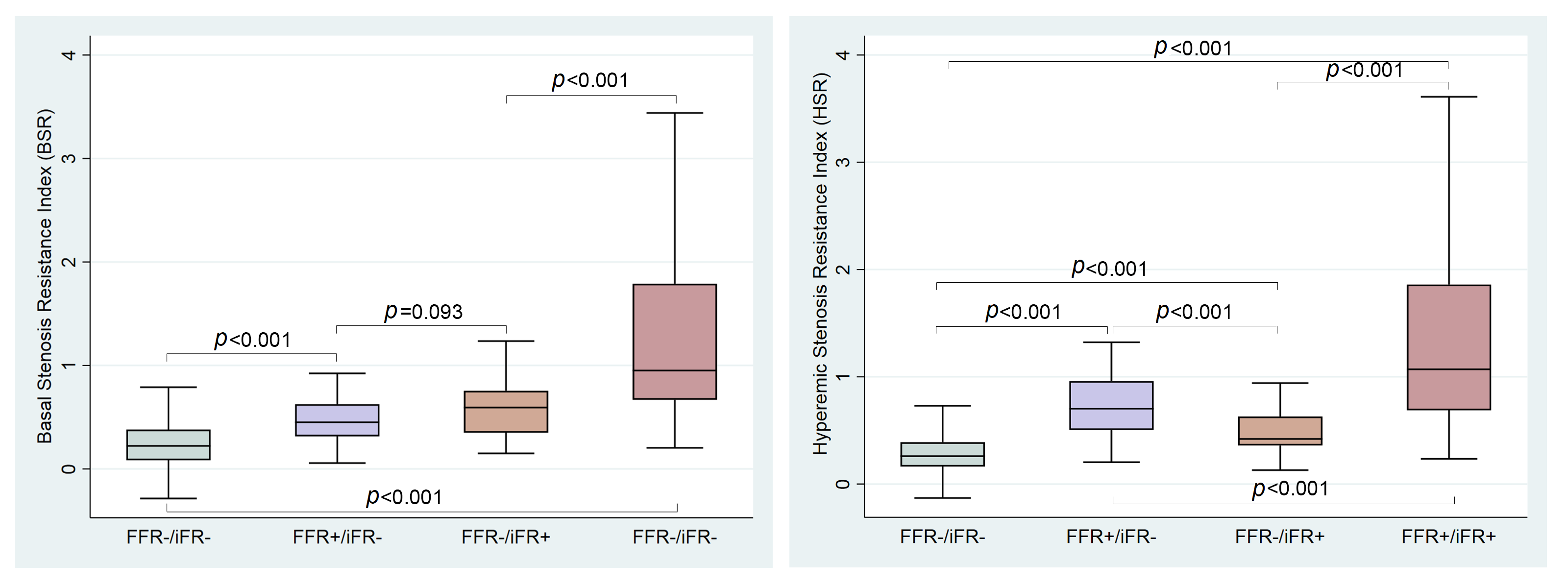

Supplement: Supplementary file 2 — Fig. S2 BSR and HSR over FFR/iFR groups (FFR/iFR fractional flow reserve/instantaneous wave-free ratio, BSR basal stenosis resistance, HSR hyperaemic stenosis resistance) [file 12471_2023_1796_MOESM2_ESM.tif]
