# Supplementary material for: Impact of stenosis resistance and coronary flow capacity on fractional flow reserve and instantaneous wave-free ratio discordance: a combined analysis of DEFINE-FLOW and IDEAL
Source: Neth Heart J. 2023 Aug 18;31(11):434–43. doi: 10.1007/s12471-023-01796-x (PMC10602988; doi:10.1007/s12471-023-01796-x)
Supplement: Supplementary file 3 — Tab. S1 Lesion and physiology characteristics per group [file 12471_2023_1796_MOESM3_ESM.docx]

**Table S1.** Lesion and physiology characteristics per group

|  | **FFR-/iFR-** | **FFR+/iFR-** | **FFR-/iFR+** | **FFR+/iFR+** | | **p-value** |
| --- | --- | --- | --- | --- | --- | --- |
| Lesions, n (%) | 363 (56%) | 55 (9%) | 42 (6%) | 187 (29%) | |  |
| Patients , n (%) | 326 (55) | 47 (8%) | 40 (7%) | 180 (30%) | |  |
| Age | 65±10 | 62±10 | 66±10 | 63±10 | | 0.02 |
| Visual diameter stenosis (DS)% | 50  [50, 60] | 60  [50, 70] | 52  [49, 66] | 70  [60, 77] | | <0.001 |
| Baseline Pa, mmHg | 98  [88, 108] | 99  [90, 107] | 93  [82, 108] | 98  [86, 109] | | 0.677 |
| Baseline Pd, mmHg | 94  [84, 105] | 91  [85, 100] | 84  [75, 97] | 77  [66, 92] | | <0.001 |
| **Pressure measurements** | | | | | | |
| iFR | 0.95  [0.93, 0.98] | 0.91  [0.90, 0.93] | 0.87  [0.84, 0.88] | 0.78  [0.61, 0.84] | | <0.001 |
| FFR | 0.88  [0.84, 0.93] | 0.75  [0.73, 0.77] | 0.84  [0.82, 0.86] | 0.67  [0.55, 0.74] | | <0.001 |
| **Flow measurements** | | | | | | |
| Baseline average peak velocity, cm/s | 15  [11, 19] | 14  [11, 17] | 16  [10, 23] | 15  [11, 20] | | 0.574 |
| Hyperaemic average peak velocity, cm/s | 34  [25, 44] | 31  [23, 44] | 29  [19, 37] | 24  [17, 34] | | <0.001 |
| CFR | 2.4  [2.0, 2.9] | 2.4  [2.0, 2.7] | 1.6  [1.4, 2.1] | 1.6  [1.3, 2.1] | | <0.001 |
| **Stenosis resistance index** | | | | | | |
| BSR, mmHg/cm/s | 0.22  [0.08-0.38] | 0.45  [0.31-0.63] | 0.59  [0.35-0.76] | 0.95  [0.67-1.79] | <0.001 | |
| HSR, mmHg/cm/s | 0.27  [0.16, 0.39] | 0.70  [0.50, 0.96] | 0.42  [0.36, 0.63] | 1.07  [0.69, 1.86] | <0.001 | |
| **Coronary flow capacity** | | | | | | |
| Normal to mildly reduced CFC, n (%) | 286 (79%) | 43 (78%) | 19 (45%) | 70 (37%) | <0.001 | |
| Moderately to severely reduced CFC, % | 77 (21%) | 12 (22%) | 23 (55%) | 117 (63%) | <0.001 | |

Continuous data are presented as mean±SD, median [Q1, Q3] or *n* (%)

*BSR* basal stenosis resistance, HSR hyperaemic stenosis resistance, *CFR* coronary flow reserve, *FFR* fractional flow reserve, *iFR* instantaneous wave-free ratio, CFC coronary flow capacity
